# A pilot investigation of the association between HIV-1 Vpr amino acid sequence diversity and the tryptophan-kynurenine pathway as a potential mechanism for neurocognitive impairment

**DOI:** 10.1186/s12985-024-02313-1

**Published:** 2024-02-23

**Authors:** Levanco Keegan Asia, Esmé Jansen Van Vuren, Zander Lindeque, Monray Edward Williams

**Affiliations:** 1 Human Metabolomics, North-West University, Potchefstroom, South Africa; 2 Hypertension in Africa Research Team (HART), North-West University, Potchefstroom, South Africa; 3 South African Medical Research Council, Unit for Hypertension and Cardiovascular Disease, North-West University, Potchefstroom, South Africa

**Keywords:** Tryptophan-Kynurenine pathway, Metabolism, Inflammation, Pathogenesis, Neuropathogenesis

## Abstract

HIV infection compromises both the peripheral and central immune systems due to its pathogenic and neuropathogenic features. The mechanisms driving HIV-1 pathogenesis and neuropathogenesis involve a series of events, including metabolic dysregulation. Furthermore, HIV-subtype-specific variations, particularly alterations in the amino acid sequences of key viral proteins, are known to influence the severity of clinical outcomes in people living with HIV. However, the impact of amino acid sequence variations in specific viral proteins, such as Viral protein R (Vpr), on metabolites within the Tryptophan (Trp)-kynurenine (Kyn) pathway in people living with HIV remains unclear. Our research aimed to explore the relationship between variations in the Vpr amino acid sequence (specifically at positions 22, 41, 45, and 55, as these have been previously linked to neurocognitive function) and peripheral Trp-Kyn metabolites. Additionally, we sought to clarify the systems biology of Vpr sequence variation by examining the link between Trp-Kyn metabolism and peripheral inflammation, as a neuropathogenic mechanism. In this preliminary study, we analyzed a unique cohort of thirty-two (*n* = 32) South African cART naïve people living with HIV. We employed Sanger sequencing to ascertain blood-derived Vpr amino acid sequence variations and a targeted LC-MS/MS metabolomics platform to assess Trp-Kyn metabolites, such as Trp, Kyn, kynurenic acid (KA), and quinolinic acid (QUIN). Particle-enhanced turbidimetric assay and Enzyme-linked immunosorbent assays were used to measure immune markers, hsCRP, IL-6, suPAR, NGAL and sCD163. After applying Bonferroni corrections (*p* =.05/3) and adjusting for covariates (age and sex), only the Vpr G41 and A55 groups was nearing significance for higher levels of QUIN compared to the Vpr S41 and T55 groups, respectively (all *p* =.023). Multiple regression results revealed that Vpr amino acid variations at position 41 (adj R^2^ = 0.049, β = 0.505; *p* =.023), and 55 (adj R^2^ = 0.126, β = 0.444; *p* =.023) displayed significant associations with QUIN after adjusting for age and sex. Lastly, the higher QUIN levels observed in the Vpr G41 group were found to be correlated with suPAR (*r* =.588, *p* =.005). These results collectively underscore the importance of specific Vpr amino acid substitutions in influencing QUIN and inflammation (specifically suPAR levels), potentially contributing to our understanding of their roles in the pathogenesis and neuropathogenesis of HIV-1.

**Supplementary Information:**

The online version contains supplementary material available at 10.1186/s12985-024-02313-1.

## Introduction

Human immunodeficiency virus (HIV) affects approximately 39 million people globally of which 37.5 million are adults and 1.5 million are children (0–14 years of age) [1]. Despite advancements in HIV treatment, twenty million people living with HIV are situated in eastern and southern Africa. HIV-1 impacts immune cells, including CD4^+^ cells, leading to the deterioration of the immune system [2]. This deterioration creates conditions conducive to the development of comorbidities [3]. HIV infection commonly results in the virus spreading into the central nervous system (CNS) of people living with HIV, consequently heightening the risk of developing HIV-associated neurocognitive disorders (HAND) [4]. However, the severity of HIV pathogenesis and neuropathogenesis may differ due to the genetic diversity of HIV [5].

While both HIV-1 and HIV-2 can contribute to HIV pathogenesis, HIV-1 is more virulent and is the predominant type causing HIV infections [6–8]. Due to the considerable genetic variability of HIV, different subtypes of HIV-1 have emerged globally, [5], each exerting distinct influences on HIV pathogenesis, neuropathogenesis, and the severity of clinical outcomes [9]. Among the HIV-1 groups, including M, N, O, and P [10–15], group M is the most prevalent globally [16]. Furthermore, group M consists of subtypes A, B, C, D, F, G, H, J, and K [10]. Subtypes B and C represent most HIV infections, accounting for 12.1% and 46.6%, respectively [5]. Subtype C is the most common in Southern Africa whereas subtype B is most common in Eastern European and Central Asia regions [5].

Clinical outcomes related to HIV progression and neuropathogenesis vary between HIV-1 subtypes [17–20]. Consequently, the functions of HIV viral proteins vary depending on the HIV subtype [21–26]. Thus, studies have investigated the effects of HIV-1 subtype-specific viral proteins such as transactivator of transcription (Tat) and glycoprotein 120 (gp120) in people living with HIV [25, 27–30]. While major viral proteins like Tat and gp120 receive significant attention, accessory viral proteins such as viral protein R (Vpr) are less frequently studied. This is in spite of research indicating Vpr’s contribution to clinical outcomes [31–34], including its association with HIV neuropathogenesis [35].

On a metabolic level, tryptophan-kynurenine (Trp-Kyn) metabolism has garnered interest because of its association with HIV-1 pathogenesis and neuropathogenesis [36, 37]. Once Trp is catabolized, it produces several downstream metabolites via the kynurenine (Kyn) pathway such as Kyn, kynurenic acid (KA), and quinolinic acid (QUIN) [36]. In the brain, Trp-Kyn metabolism occurs mainly in infiltrating macrophages and resident microglial cells [38, 39]. Given that HIV infects macrophages that traverse the blood-brain barrier, a potential link between the Trp-Kyn metabolism and HIV neuropathogenesis might exist. Furthermore, dysregulated inflammation is a major driver of disease progression [40, 41], HIV persistence [42], neuropathogenesis, and the development of milder forms of HAND in the modern-ART era [43, 44]. Dysregulated inflammation is observed in plasma and cerebrospinal fluid (CSF) of people living with HIV [41, 45, 46]. This inflammation is closely associated with metabolic changes, especially in Trp metabolism [47]. Furthermore, the enzyme, IDO-1, which is responsible for Trp degradation into Kyn is upregulated by inflammatory molecules and cytokines like interferon-gamma (IFN-y) [48, 49]. Subsequently, Trp degradation via the Trp-Kyn pathway results in metabolites which may have excitatory and inhibitory properties [50, 51]. Thus, these excitatory properties play a role in the HIV infection in the CNS [52, 53]. The HAND population often exhibits lowered Trp and Kynurenine/Tryptophan (Kyn/Trp) ratio levels in plasma [47].

Although there is an association between Trp-Kyn metabolism and HIV neuropathogenesis [36], the association between Trp-Kyn metabolism and HIV-1 viral proteins are unclear; especially the association between HIV-1 Vpr sequence variation and the Trp-Kyn metabolism. In a previous systematic review conducted by our group, Vpr’s association with clinical outcomes in people living with HIV was evident [22]. However, to our best knowledge, this is the first study to investigate the influence of Vpr amino acid sequence variation on the Trp-Kyn metabolism. Thus, we aimed to assess whether Vpr amino acid sequence variation, especially the neurological-associated amino acids at positions 22, 41, 45, and 55, associates with metabolites of Trp-Kyn metabolism in a South African cohort.

## Methods

### Study participants

The Prospective Urban and Rural Epidemiology (PURE) study focuses on cardiovascular diseases (CVD) in people living with HIV and the underlying mechanism that can contribute to the development of CVD such as inflammation, in individuals from 20 countries with varying income levels, encompassing high, middle, and low-income nations [54]. As a subset of this extensive research, men and women aged 30 and above of African descent were enlisted from both urban and rural regions in the North-West Province of South Africa. The participants were excluded if they had chronic medication use and existing chronic conditions. Baseline data collection, encompassing *n* = 2,010 participants, was voluntarily conducted in 2005. Subsequent follow-up data was gathered in 2010 (*n* = 1,288) and 2015 (*n* = 923). Participants were re-diagnosed in 2010 to confirm their HIV status. Therefore, individuals who screened positive for HIV-1 and were treatment-naïve at the time of data collection in 2010 were included in this study (*n* = 103). HIV-1 sequencing was successful for only *n* = 32 people living with HIV; thus, only these were included in the subsequent analysis. This selection allowed us to explore metabolic profiles without the interference of antiretroviral treatment (ART), given that ART is recognized for its impact on metabolic profiles in people living with HIV [55]. The study’s protocol received approval from the Health Research Ethics Committee of the North-West University in South Africa under the reference numbers NWU-00106-22-A1 and NWU-00106-22-A1-01.

### HIV status

Before establishing the HIV status of the participants, they underwent counselling sessions provided by trained counsellors. The HIV status was determined using the First Response rapid HIV card test, manufactured by Premier Medical Corporation Limited in Daman, India, in accordance with the protocol established by the South African Department of Health. As a confirmation step, an SD BIOLINE HIV ½ 3.0 card test from Standard Diagnostics, INC in Korea was used. Participants who received a positive result were provided with post-counselling and were then referred to the nearest clinic or hospital for further assessment. At the clinic or hospital, their CD4^+^ cell count was analysed using the flow cytometric method with the Beckman COULTER EPICS XlTM machine from Fullerton, USA.

### Analysis of metabolites in blood samples

Fasting blood samples were collected and were centrifuged at 2000 × g for 15 min at a temperature of 10 °C, all within 2 h of collection. After centrifugation, the samples were transferred into microfuge tubes, promptly frozen by placing them on dry ice, and then stored at -80 °C in the laboratory until they were ready for analysis. In cases where samples were collected from rural areas, they were also snap-frozen on dry ice but stored at -18 °C for a maximum of five days until they could be transported to the laboratory. Upon arrival, they were then stored at -80 °C for subsequent analysis.

We investigated the Trp-Kyn metabolism metabolites, specifically Trp, Kyn, Kyn:Trp ratio (in direct measure of IDO activity), KA, and QUIN, due to their potential involvement in the pathophysiology of Vpr-mediated neuropathogenesis and HAND, as discussed in scientific literature [36, 38, 39, 47–51]. These metabolic profiles were determined using high-performance liquid chromatography (HPLC) with tandem mass spectrometry (MS/MS) as described in the following sections.

#### Chemicals

Kynurenic acid-d5 (2.5 mg), L-Kynurenine-d4 [2-aminophenyl-3,5-d2] (5 mg), 2,3-pyridinedicarboxylic acid-d3 (Major) (1 mg) were purchased from TRC Research Chemical. D-Tryptophan (Indole-D5, 98%) (50 mg) was purchased from Cambridge Isotope Laboratories. D-Kynurenine free base (25 mg), 2,3-dicarboxylic acid, 99% (25 g), D-tryptophan, = 98.0% (HPLC) (1 g) was purchased from MERCK. UPLC water and acetonitrile, 99%, was purchased from Honeywell. Formic acid for LC-MS was purchased from Merck.

#### Sample preparation for targeted metabolomics

Trp, Kyn, Kyn/Trp ratio, QUIN, and KA were quantified by liquid chromatography–tandem mass spectrometry (LC-MS/MS; Agilent 1200 series HPLC system) using a targeted approach. Proteins were precipitated from HIV plasma samples by adding 300 µL ice-cold acetonitrile to 100 µL HIV plasma samples in addition to 100 µL internal standard mixture (10 ppm, Kynurenic acid-d5, L-Kynurenine-d4 [2-aminophenyl-3,5-d2], 2,3-pyridinedicarboxylic acid-d3 (Major), D-Tryptophan (Indole-D5, 98%)) [56]. Matrix-appropriate external calibrators were prepared similarly. External calibrators were spiked with Kyn, QUIN, and Trp, which were serially diluted to create a calibration curve. Samples were vortexed and incubated on ice for 10 min to facilitate protein precipitation. Samples were then centrifuged at 12 000x g for 10 min. The supernatant was collected, dried under nitrogen gas then stored at -80 °C until analysis. For analysis, samples were removed from the − 80 °C freezer and left at room temperature to equilibrate. The samples were then re-dissolved in 50% HPLC water: 50% ACN, left for 30 min at room temperature and then vortexed. The resuspended sample was transferred to a glass vial with a vial insert. Samples were then analyzed on the LC-MS/MS. Metabolic profiles were measured via multiple reaction monitoring (MRM) using electrospray ionization mass spectrometry in both positive and negative modes. The MRM transitions and chromatographic conditions on the LC-MS/MS were optimized (with the help of commercial standards) to detect and quantify the target metabolites accurately. The ratio of Kyn to Trp was used for the estimation of IDO activity.

#### LC-MS/MS analyses

A targeted LC-MS/MS analysis was performed on HIV-positive plasma samples using a 1200 series HPLC system coupled to a 6470-triple quadrupole-mass spectrophotometer (Agilent Technologies). The chromatographic separations were achieved by injecting 1µL sample on an Acquity UPLC CSH C18 1.7 μm 2.1 × 100 mm column, kept at 80 °C. Mobile phases consisted of HPLC water (solvent A) and acetonitrile (solvent B) with both containing 0.1% formic acid.

For the positive polarization, the chromatographic gradient was set up as t = 0, 1% mobile phase B; t = 1, 2% mobile phase B; t = 2, 5% mobile phase B; t = 3, 10%mobile phase B; t = 4, 40% mobile phase B; t = 5, 50% mobile phase B; t = 6, 60% mobile phase B; t = 7, 80% mobile phase B; t = 8, 100% mobile phase B; t = 10, 1% mobile phase B; lastly, 100% mobile phase B for 5 min (post-run) to ensure that all analytes are washed out of the column for the next sample analysis. The flow rate was always 0.2 mL/min except at t = 1 until t = 2, 0.1 mL/min. Source parameters were set as capillary voltage of 3500 V; nitrogen gas at a flow rate of 5 L/min at 300 °C; and nebulizer pressure of 45 psi. MRM parameters were set up for all compounds (Supplementary Table 1).

For the negative polarization, the chromatographic gradient was set up as t = 0, 1% mobile phase B; t = 2, 1% mobile phase B; t = 3, 37% mobile phase B; t = 4, 40%mobile phase B; t = 5, 45% mobile phase B; t = 6, 50% mobile phase B; t = 7, 100% mobile phase B; t = 8, 1% mobile phase B; lastly, 100% mobile phase B for 5 min (post-run) to ensure that all analytes are washed out of the column for the next sample analysis. The flow rate was 0.1 mL/min at t = 0; t = 2; t = 3 and 0.2mL/min at t = 4; t = 5; t = 6; t = 7; t = 8. Source parameters were set as capillary voltage of 4000 V; nitrogen gas at a flow rate of 8 L/min at 250 °C; and nebulizer pressure of 20 psi. MRM parameters were set up for all compounds (Supplementary Table 1).

### Analysis of immune markers in blood samples

Samples were prepared as described above (*Sect. 2.3*). We selected specific immune markers for investigation, including soluble urokinase plasminogen activator receptor (suPAR), interleukin (IL) 6, high-sensitivity C-reactive protein (hsCRP), soluble CD163 (sCD163), and neutrophil gelatinase-associated lipocalin (NGAL). These markers were chosen based on their potential relevance to Vpr-mediated neuropathogenesis and HAND pathophysiology, as indicated in scientific literature [22, 57–59]. To measure suPAR levels, plasma samples with EDTA were used, and the suPARnostiC® ELISA kit from ViroGates in Copenhagen, Denmark, was employed. Particle-enhanced turbidimetric assay was used to analyze hsCRP levels (Cobas Integra 400 plus (Roche Diagnostic, Basel, Switzerland)), while IL-6 was determined in plasma through the electrochemiluminescence immunoassay method using an Elecsys 2010 (Roche, Basel, Switzerland) apparatus. For sCD163 and NGAL, ELISA assays (R&D systems DuoSet) were conducted following the manufacturer’s instructions, and all samples were analyzed in duplicate. The intra- and inter-assay coefficients of variation for all tests fell within acceptable ranges of < 8% and < 10%, respectively.

### Viral protein analysis

RNA was isolated from 200 µL of prepared plasma using the Quick-RNA™ Viral Kit from Zymo Research. The total RNA was then subjected to reverse transcription-polymerase chain reaction (PCR) using the ProtoScript® II First Strand cDNA Synthesis Kit from New England Biolabs, and the resulting DNA was prepared for subsequent PCR analysis. For the amplification of the Tat exon 1/Vpr region (HXB2 position 4900–6351), a primer pair consisting of Vif-1 (5’GGGTTTATTACAGGGACAGCAGAG) and CATH-4R (5’-GTACCCCATAATAGACTGTGACC) was employed. The PCR amplification process involved an initial denaturation step at 94 °C for 2 min, followed by 40 cycles of denaturing at 94 °C for 30 s, annealing at 60 °C for 30 s, extension at 68 °C for 2 min, and a final extension step at 68 °C for 10 min. Following PCR amplification, purification of all PCR products was carried out using the Nucleospin® Gel and PCR clean-up kit in accordance with the manufacturer’s instructions (Machery-Nagel GmbH & Co.KG, Germany). Subsequently, all PCR products were subjected to sequencing using the BigDye Terminator v.3.1 Cycle Sequencing Ready Reaction Kit from ThermoFisher Scientific and were analyzed using the ABI Prism 3130xl automated DNA sequencer from Applied Biosystems, located in Foster City, CA. To analyze the obtained sequences, the GeneStudio™ Professional sequence analysis software (Version 2.2) was utilized. The nucleotide sequences were translated into amino acid sequences using the Expasy translate method [60], and the key mutations in the Vpr region were identified and highlighted for further examination. Sequences are available in GenBank under the accession numbers OR621303- OR621349.

### Statistical analysis

All analyses were conducted using SPSS (version 27, IBM, USA). *P*-values were considered statistically significant for all analyses at less than 0.05. All variables were assessed for normality by the visual inspection of QQ plots using descriptive statistics. Data distribution for age, the metabolites Kyn/Trp ratio, QUIN and KA and the immune markers NGAL and sCD163 were skewed. Therefore, the data of variables that were skewed were log-transformed before statistical analyses. After log transformation, all data presented acceptable skewness and kurtosis values within the range of -2 and 2.

As the primary aim, we wanted to evaluate whether specific metabolites levels could be related to single amino acid variants. Therefore, we stratified participants into groups: I22 vs. L22, N/S41 vs. G41, Y45 vs. H45, and A55 vs. T55. At position 41, we compared G vs. N and G vs. S, respectively. Chi-squared tests were used to test group differences for sex, smoking, alcohol use and locality between Vpr amino acid variants. Independent sample T-tests determined differences in study characteristics (age, CD4^+^ count, BMI) and metabolite levels between Vpr amino acid variants. A Bonferroni correction was accounted for the number of metabolites tested (α/*n* =.05/5 = 0.01) in the primary aim. A Pearson correlation analysis was used to determine covariates by determining correlations between sociodemographic variables (age, sex, smoking status, BMI, alcohol use, and locality), and specific metabolites. Analyses of covariance (ANCOVA) were performed with levels of metabolites as the dependent variables to compare the metabolite levels between Vpr variants, adjusting for covariates. A Bonferroni correction was accounted for the number of tests (α/*n* =.05/3 = 0.017) within the model (ANCOVA). Multiple regression analysis was used to determine associations between Vpr amino acid variants and metabolite levels after adjusting for potential covariates.

As a secondary aim, we wanted to determine the association between peripheral immune markers and metabolites. Pearson correlations were used to determine correlations between metabolite and immune marker levels/ CD4^+^ count. A Bonferroni correction was accounted for the number of immune markers tested (α/*n* =.05/5 = 0.01) in the secondary aim. Thereafter, using the enter method, multiple regression analysis was used to determine associations between immune marker and metabolites levels after adjusting for potential covariates.

## Results

### Study characteristics

This pilot study encompassed a sample of *n* = 32 treatment-naïve South African subtype C participants, with an average age of 48.08 (± 7.066) years. Only 25% of the participants were males. While the primary study did not record any viral load data, it should be noted that all participants were treatment-naïve at the time of sample collection. CD4^+^ count data was available for 53% of the participants, showing a mean value of 282.76 (± 161.024) cell/mm^3^. Participants had a mean BMI of 26.64 (± 10.2). Data on smoking status and alcohol consumption were available for 94% (*n* = 30) of the participants, with 56% and 60% of these participants being current or former smokers and alcohol consumers, respectively. Approximately half of the participants were recruited from rural regions in South Africa (*n* = 18, 56%). When participants were stratified according to Vpr amino acid variants (Table 1), no significant differences were observed for any demographic variables (age, sex, CD4^+^ count, BMI, smoking status, alcohol use and locality) (all *p* >.05). After determining correlations between sociodemographic variables and specific metabolites, it was indicated that, no correlations existed for any of the considered variables (age, sex, smoking status, alcohol use, BMI and locality). Nevertheless, we opted to incorporate sex and age as covariates in the pertinent analyses as will be described below, given their established influence on HIV pathogenesis [60, 61].

### Metabolite levels between Vpr amino acid variants

We aimed to examine whether we could link metabolite levels to specific amino acid variants (Table 1). We stratified participants according to Vpr amino acid variants at position 22 (*n* = 29), 41 (*n* = 27), 45 (*n* = 30), and 55 (*n* = 28). After applying Bonferroni corrections of *p* =.05/5, none of the metabolite levels showed significant differences between the Vpr variants at positions 22, 41 or 45. However, QUIN levels were nearing significance for higher levels in the A55 group compared to the T55 group (*p* =.022) (Table 1). After applying Bonferroni corrections of *p* =.05/3, this result remained unchanged in the Vpr 55 group (*p* =.023) after adjustment for covariates (age and sex). Additionally, following the adjustment for age and sex, QUIN levels were nearing significance for higher levels (*p* =.023) in the G41 group when compared to the S41 group.

**Table 1:** Characteristics of HIV-positive participants and metabolite levels when participants stratified according to specific amino acids variants

	Position 22 (*n* = 29)		P value	Position 41 (*n* = 27)			P value	Position 45 (*n* = 30)		P value	Position 55 (*n* = 28)		P value
Amino acid	I	L		G	N	S		Y	H		A	T	
N (%)	16 (55%)	13 (45%)	-	15 (55%)	6 (22%)	6 (22%)	-	18 (60%)	12 (40%)	-	7 (25%)	21 (75%)	-
Age in years, mean (SD)	47.31 (6.23)	49.08 (8.43)	0.523	47.47 (4.9)	46.83 (7.6)	45.7 (8.7)	0.822/ 0.548	48.44 (6.5)	48.1 (8.4)	0.895	49.43 (6.6)	47.57 (6.6)	0.525
Sex, female N (%)	10 (63%)	11 (85%)	0.624	10 (67%)	5 (83.3%)	4 (6707%)	0.733	15 (83%)	9 (75%)	0.576	7 (64%)	27 (84%)	0.145
CD4 ^+^, mean (SD)	274.50 (119.21)	313.71 (139.52)	0.662	272.2 (191)	213.7 (74.2)	367 (222.6)	0.564/ 0.513	287.71 (164.3)	241. 8 (125.4)	0.535	150.5 (72.8)	330.92 (152)	0.132
Body Mass index (BMI)	235.5 (16)	223 (34)	0.532	22.3 (3.4)	23 (8.4)	21.5 (2.5)	0.747/ 0.640	23 (4.8)	23.5 (5.8)	0.811	20.5 (4)	24.2 (5.4)	0.118
Smoker	9 (56%)	7 (64%)	0.701	7 (47%)	3 (50%)	6 (100%)	0.167	9 (56%)	8 (67%)	0.576	6 (86%)	11 (55%)	0.148
Alcohol use	9 (56%)	5 (45%)	0.581	6 (40%)	2 (33%)	5 (83%)	0.253	7 (44%)	6 (50%)	0.743	3 (42%)	11 (55%)	0.580
Locality, rural N (%)	6 (38%)	9 (69%)	0.089	10 (67%)	4 (67%)	3 (50%)	0.757	10 (56%)	7 (58%)	0.880	4 (57%)	11 (52%)	0.827
Trp, ug/mL	0.16 (0.06)	0.13 (0.07)	0.223	0.17 (0.07)	0.13 (0.08)	0.13 (0.04)	0.303/ 0.226	0.15 (0.07)	0.15 (0.06)	0.710	0.15 (0.07)	0.15 (0.06)	0.822
Kyn, ug/mL	0.0087 (0.003)	0.0088 (0.003)	0.904	0.01 (0.002)	0.01 (0.003)	0.008 (0.004)	0.587/ 0.254	0.009 (0.002)	0.009 (0.001)	0.646	0.009 (0.003)	0.008 (0.003)	0.589
Kyn/Trp ratio	0.062 (0.027)	0.085 (0.042)	0.106	0.07 (0.034)	0.089 (0.045)	0.0586 (0.032)	0.290/ 0.418	0.068 (0.036)	0.071 (0.032)	0.739	0.074 (0.038)	0.061 (0.026)	0.375
QUIN, ug/mL	0.0013 (0.0009)	0.0015 (0.0009)	0.543	0.0017 (0.0009)	0.0013 (0.0004)	0.0009 (0.0007)	0.368/.*073*	0.0016 (0.0009)	0.0012 (0.0007)	0.157	0.002 (0.0012)	0.001 (0.0005)	**0.022**
KA, ug/mL	0.000014 (00002)	0.00002 (0.00003)	0.115	0.00003 (0.00003)	0.000008 (0.00003)	0.000007 (0.00003)	0.076/ 0.126	0.00002 (0.00003)	0.00001 (0.00001)	0.725	0.00001 (0.00001)	0.00002 (0.00003)	0.666

Data represented as mean (SD) or N (%). Logarithmically transformed variables has been back transformed for presentation purposes. At position 41, we compared G vs. N and G vs. S indicated by p-value/p-value, respectively. Data were unavailable for 2 participants in the L22 and Y45 groups and 1 participant in the T55 group (alcohol use and smoking)

Further, we aimed to evaluate the relationship between Vpr amino acid sequence variation and metabolite levels while adjusting for covariates (age and sex) as shown in Table 2. Here, variation at position 41 (between G41 and S41) showed significant associations with QUIN levels (*p* =.023). When investigating the Vpr amino acids at position 55, variation at this position (between A55 and T55) demonstrated significant associations with QUIN levels after (*p* =.023). No significant associations were detected for the other Vpr amino acid signatures or metabolites.

**Table 2:** Multiple regression analyses of the association between Vpr group and QUIN, including covariates

Vpr group	Adj R^2^	β	CI	p
**41**	0.204	0.505	0.133 to 1.544	0.023
**55**	0.126	0.444	0.102 to 1.257	0.023

Model 1: adjusted for demographics (age and sex)

### Correlation between immune markers and metabolites in the specific vpr groups

**Fig. 1:**
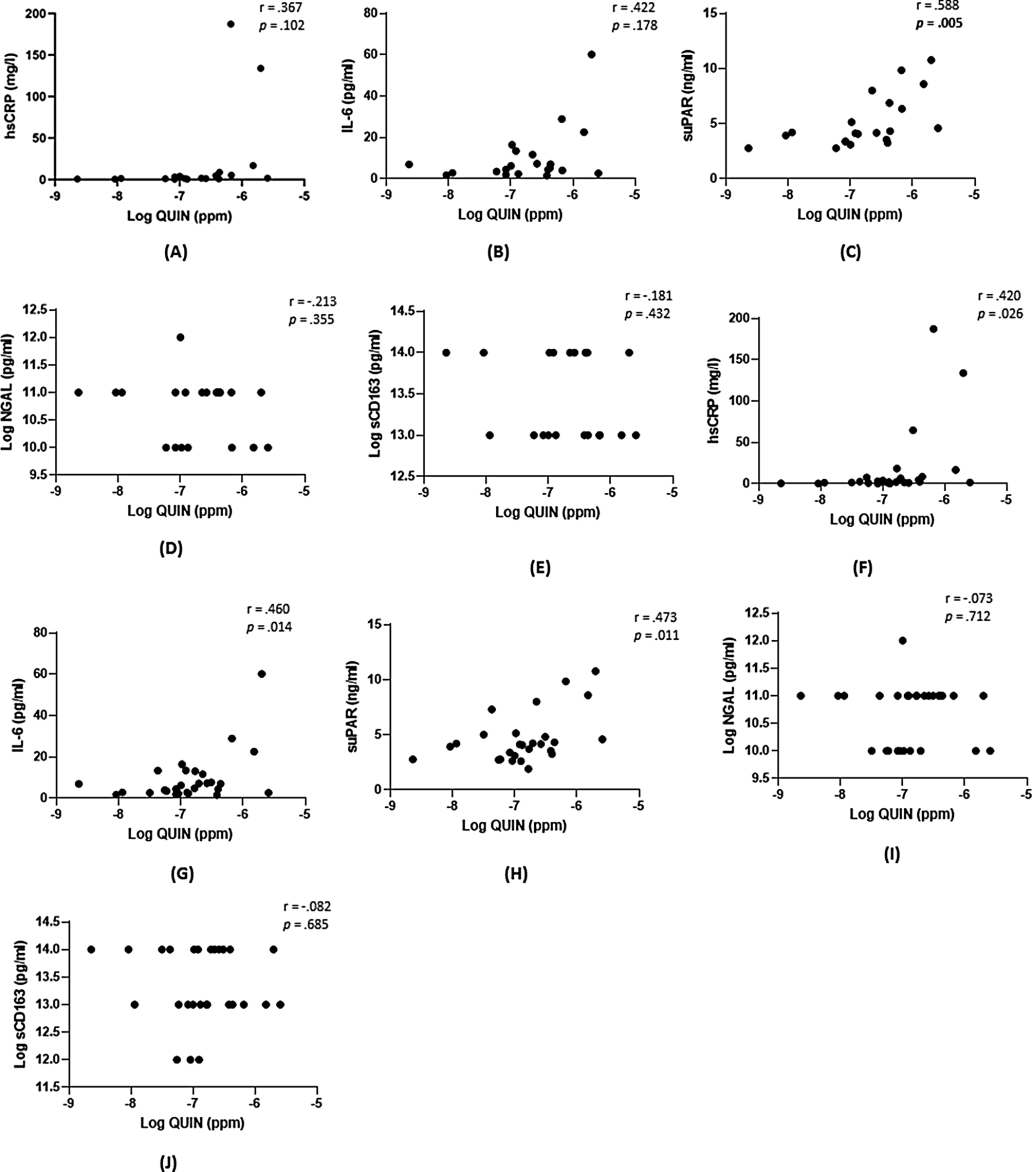
Scatter plot depicting the Pearson correlation between QUIN and immune marker levels. (**A**-**E**) correspond to the Pearson correlation within the Vpr 41 group and (**F**-**J**) correspond to the Pearson correlation within the Vpr 55 group. QUIN levels: PPM = ug/mL.

Given the significant associations between QUIN levels and variation at Vpr amino acid position 41 and 55, we sought to investigate potential correlations between QUIN and immune markers in these respective groups. Following the application of a Bonferroni correction *(p* =.05/5), we observed a significant positive correlation between suPAR and QUIN levels in the Vpr 41 group (*p* =.005) (Fig. 1C). In the Vpr 55 group, QUIN was not associated with any of the investigated immune markers (all *p* >.01) (Fig. 1F and J). Subsequently, we aimed to assess the association between QUIN and suPAR levels while adjusting for covariates. Even after adjusting for covariates, including age and sex, QUIN was significantly associated with suPAR within Vpr 41 group (adj R^2^ = 0.356, β = 0.664; *p* =.003). Given the absence of viral load data for the entire cohort, adjustment for this variable was not possible. However, CD4^+^ count data was accessible for 53% of the participants. Consequently, we sought to explore the extent to which these disease characteristics may have contributed to QUIN levels in our study. In the overall cohort with available CD4^+^ data (*n* = 17), QUIN levels were negatively correlated with CD4^+^ count (*r* = −.711, *p* =.001).

## Discussion

Our study encompassed three main findings. Firstly, among the various Vpr amino acid variants and metabolites examined, only the Vpr G41 and A55 group was nearing significantly higher levels of QUIN when compared to the Vpr S41 and T55 variants, respectively. Secondly, amino acid variation at positions 41 and 55 were found to be significantly associated with QUIN levels. Lastly, in the Vpr G41 group, QUIN correlated with higher suPAR levels. These results collectively underscore the importance of specific Vpr amino acid substitutions in influencing QUIN and specifically, suPAR levels, potentially contributing to our understanding of their roles in the pathogenesis and neuropathogenesis of HIV-1.

The findings of this study report significant associations between QUIN levels and Vpr variation at positions 41 (between G41 and S41) and 55 (between A55 and T55), respectively. These findings may indicate that Vpr variation at these specific positions could increase QUIN levels. Indeed, we showed that the Vpr G41 and A55 variant showed higher levels of QUIN compared to the S41 (*p* =.023) and T55 groups (*p* =.023), respectively. The higher QUIN levels could potentially be indicative of negative clinical outcomes, as peripheral and CSF QUIN levels have previously been associated with neurological outcomes in psychiatric disorders, including HAND [61, 62]. Although QUIN cannot freely cross the BBB [63], it is a product of 3-hydroxykynurenine, which can cross the blood-brain barrier freely. Additionally, 3-hydroxykynurenine is a downstream metabolite of tryptophan, which can also readily cross the blood-brain barrier [64]. As a result, the production of QUIN in the brain may persist, maintaining its neuroexcitatory properties [51]. During immune activation in the CNS, over 98% of the Kyn and QUIN present in the brain are produced within the CNS [65]. However, studies have demonstrated that human monocytes and monocyte-derived macrophages can produce up to 19 times more QUIN than activated microglia [66]. In line with this, the synthesis of QUIN by microglia in patients with epilepsy was approximately only 15% of the QUIN produced by monocyte-derived macrophages retrieved from brain tissue [67]. Lastly, it’s important to highlight that QUIN concentrations consistently tend to be higher in the bloodstream compared to the CNS across various pathological conditions, with blood-to-cerebrospinal fluid (CSF) ratios of 14:1 in humans, 19:1 in rodents, and even up to 52:1 in nonhuman primates [68]. This observation is also pertinent to the CNS because infiltrating activated macrophages could potentially serve as the primary source of QUIN during brain inflammation. Therefore, an investigation into systemic QUIN levels may provide valuable insights into how these levels may be reflected in the CNS. These findings may have translation value to other health conditions. Other studies have also identified associations between plasma Trp-Kyn metabolites and various health conditions, including the presence of depression [69], HIV-related gut microbiota alterations, and gut-adipose tissue [70]. Moreover, these metabolites have been shown to predict poor CD4 ^+^ T-cell count recovery and increased mortality among individuals with HIV infection. Additionally, they may play a role in discriminating modest detection of active tuberculosis in adults with HIV [71, 72].

A study investigating treatment experienced people living with HIV found that the A55 amino acid to be linked with adverse neurological outcomes [73]. According to Dampier and colleagues, people living with HIV who possess the A55 amino acid variant tend to have higher global deficit scores [73]. However, an opposing viewpoint has been proposed, suggesting positive clinical outcomes. A study associated the Vpr A55 amino acid variant with a lower plasma viral load in treatment naïve people living with [74]. Our findings suggest that the A55 variant, by means of the Trp-Kyn metabolism may be associated with negative clinical outcomes by enhancing inflammation as QUIN correlated with suPAR in participants with this variant only. However, further studies are needed to investigate this variant in larger cohorts to validate the findings reported here.

Previous studies have found that the G41 variant is associated with positive clinical outcomes and is observed in treatment experienced long-term non-progressor people living with HIV and non-stroke control groups [75, 76]. Furthermore, McMullen and colleagues found that the S41 variant is associated with lower CD4^+^ counts and ischemic stroke [75]. Conversely, an earlier study by Dampier and colleagues found that the S41 variant in treatment-experienced participants was associated with positive clinical outcomes, as participants exhibited decreased neurocognitive deficits [73], aligning with our findings. Although limited studies have investigated Vpr amino acid variation at position 41, it is evident that there is no clear consensus regarding the influence of G41/S41 amino acids on clinical outcomes in people living with HIV. However, our findings suggest that G41 may be considered a high-risk amino acid in people living with HIV since the G41 group had higher QUIN levels which correlated with suPAR in this group only, which are believed to be characteristic of negative clinical outcomes [51, 77]. It is possible that amino acid 41 is located within the second alpha-helix (38–50), a structurally significant region for key mechanisms such as the regulation of apoptosis, subcellular transport [78] and virion incorporation of Vpr [79]. Consequently, modifying this configuration and changing the amino acids at this position could potentially disrupt the arrangement of the Vpr alpha helices, thus affecting its functional potential. However, further investigation using modelling and structural techniques is needed to assess the potential functional implications of different amino acids at these positions. While QUIN is known to be neuroexcitatory and linked to worsened neurological outcomes [51], it is important to note that higher suPAR levels have also been associated with decreased neurological performance in people living with HIV [57, 80]. Considering the association of suPAR with neurological outcomes, it’s worth considering that suPAR may play a role in Trp-Kyn metabolism as an alternate mechanism in the development of HAND. suPAR is known to contribute to an elevated inflammatory environment, and a proinflammatory milieu can stimulate Trp-Kyn metabolism, particularly through the activity of IDO-1. This suggests that suPAR may play a potential role in the breakdown of tryptophan, leading to the subsequent production of the downstream product QUIN [48, 49]. These findings imply that the inflammatory-associated marker suPAR could further contribute to neurotoxicity due to its significant correlation with the neurotoxic metabolite QUIN. Additionally, suPAR has been shown to be a novel, independent predictive marker of myocardial infarction in people living with HIV [81] which may further contribute to the continuous increase in mortality rates in people living with HIV [82, 83].

While our investigation focused on peripheral metabolite levels, we believe that these findings could be relevant to the activity of these metabolites in the CNS. The activation of the Trp-Kyn metabolism in the brain due to cytokine stimulation may have parallels in peripheral tissues [62]. In theory, assessments of peripheral metabolites such as QUIN or KA could indirectly offer insights into the conditions within brain tissue [62]. Furthermore, in a recent systematic review conducted by our group comprising 22 studies investigating Vpr amino acid substitutions and clinical outcomes, 10 of the studies included treatment-experienced participants, 8 did not report treatment status, and only 4 studies reported on participants who were treatment-naïve [22]. Therefore, our study provides insight into a relatively scarce population of treatment-naïve individuals within the HIV population.

## Limitations

This study possesses several noteworthy limitations that deserve attention. First, the sample size was restricted, potentially influencing the reported findings. Therefore, it is essential to interpret our findings considering this limitation. Due to the limited sample size, there is need for future research to confirm our results with larger study cohorts. While we consider this study as preliminary, it provides valuable insights into the link between Vpr sequence variations in a cohort of treatment-naïve individuals, offering a clearer understanding of the metabolic profile without the potential confounding effects of combination cART. Second, we also examined peripheral metabolic and immune markers to gain insights into their potential role in central nervous system (CNS) function [45, 84]. Furthermore, other peripheral markers, including neurofilament light (NFL), which were not investigated in this study, might have offered additional insights into the neuropathology of HIV-1. Previous studies have established a significant correlation between peripheral NFL levels and those within the CNS. Plasma NFL has been shown to detect both severe and subclinical neuronal injury in HIV [85]. Therefore, incorporating this marker would have been ideal for fully appreciating the translational value of our findings within the CNS. Lastly, studies have identified that plasma Trp-Kyn metabolites predict poor CD4 ^+^ T-cell count recovery and increased mortality among individuals with HIV infection. Seeing as CD4 ^+^ count and viral load, were unavailable for a substantial subset of participants in our study, we could not consider these variables as covariates. In a subset of participants with CD4 ^+^ count data, we observed a significant negative correlation between CD4 ^+^ count and QUIN levels. This suggests that CD4^ +^ count may have influenced the reported levels of QUIN in this study. However, further investigation is required, and it is essential to acknowledge that this limitation may have impacted our findings.

## Conclusion

Here we report evidence that the Vpr amino acid sequence variations may have an influence on metabolic and inflammatory systems, particularly in the Trp-Kyn metabolism. The associations between these sequence variations and Trp-Kyn metabolite levels, alongside the significant correlations with immune markers, provide insights into potential mechanisms contributing to the HIV-1 pathogenesis and potentially neuropathogenesis. This research underscores the importance of understanding the genetic nuances of HIV and their broader systemic implications, emphasizing the need for continued exploration in this area to better understand the pathogenesis and neuropathogenesis in of HIV-1 people living with HIV.

### Electronic supplementary material

Below is the link to the electronic supplementary material.

Supplementary Material 1: LC-MS/MS MRM parameter setup

## Data Availability

All data generated or analysed during this study are included in this published article [and its supplementary information files].
