# Supplementary material for: A pilot investigation of the association between HIV-1 Vpr amino acid sequence diversity and the tryptophan-kynurenine pathway as a potential mechanism for neurocognitive impairment
Source: Virol J. 2024 Feb 23;21:47. doi: 10.1186/s12985-024-02313-1 (PMC10893664; doi:10.1186/s12985-024-02313-1)
Supplement: Supplementary file 1 — Supplementary Material 1: LC-MS/MS MRM parameter setup [file 12985_2024_2313_MOESM1_ESM.docx]

**Supplementary Table 1:** LC-MS/MS MRM parameter setup

| Compound name | Precursor ion (m/z) | Product ion (m/z) | Dwell time | Fragmentor voltage (V) | Collision energy (eV) | Polarization |
| --- | --- | --- | --- | --- | --- | --- |
| L-kynurenine-d4 | 213.1 | 196 | 200 | 80 | 8 | Positive |
| D-tryptophan-d5 | 210.1 | 192 | 200 | 75 | 8 | Positive |
| D-kynurenine | 209.1 | 192 | 200 | 75 | 8 | Positive |
| D-tryptophan | 205.1 | 188 | 200 | 70 | 8 | Positive |
| Kynurenic acid-d5 | 195.1 | 149 | 200 | 91 | 24 | Positive |
| Quinolinic acid-d3 | 171 | 81.1 | 200 | 75 | 28 | Positive |
|  |  |  | 200 |  |  |  |
| L-kynurenine-d4 | 211.1 | 193 | 200 | 75 | 8 | Negative |
| D-tryptophan-d5 | 208.1 | 120.1 | 200 | 128 | 20 | Negative |
| Kynurenic acid-d5 | 193.1 | 149.1 | 200 | 101 | 16 | Negative |
| Kynurenic acid | 190 | 144 | 200 | 86 | 20 | Negative |
| Quinolinic acid-d3 | 169 | 125 | 200 | 70 | 8 | Negative |
| Quinolinic acid | 166 | 122 | 200 | 70 | 8 | Negative |
